# Body size trajectories and cardio‐metabolic resilience to obesity

**DOI:** 10.1111/nbu.12350

**Published:** 2018-10-15

**Authors:** W. Johnson

**Affiliations:** ^1^ Loughborough University Loughborough UK

**Keywords:** body mass index, body size trajectories, cardio‐metabolic health, healthy obesity, life course epidemiology, resilience

## Abstract

Individuals with obesity do not represent a single homogenous group in terms of cardio‐metabolic health prospects. The concept of metabolically healthy obesity is a crude way of capturing this heterogeneity and has resulted in a plethora of research linking to future outcomes to show that it is not a benign condition. By contrast, very few studies have looked back in time and modelled the life course processes and exposures that explain the heterogeneity in cardio‐metabolic health and morbidity and mortality risk among people with the same body mass index (BMI) (or waist circumference or percentage body fat). The aim of the Medical Research Council New Investigator Research Grant (MR/P023347/1) ‘Body size trajectories and cardio‐metabolic resilience to obesity in three United Kingdom birth cohorts’ is to reveal the body size trajectories, pubertal development patterns and other factors (*e.g*. early‐life adversity) that might attenuate the positive associations of adulthood obesity makers (*e.g*. BMI) with cardio‐metabolic disease risk factors and other outcomes, thereby providing some degree of protection against the adverse effects of obesity. This work builds on the Principle Investigator's previous research as part of the *Cohort and Longitudinal Studies Enhancement Resources* initiative and focuses on secondary data analysis in the nationally representative UK birth cohort studies (initiated in 1946, 1958 and 1970), which have life course body size and exposure data and a biomedical sweep in adulthood. The grant will provide novel evidence on the life course processes and exposures that lead to some people developing a cardio‐metabolic complication or disease or dying while other people with the same BMI do not. This paper details the grant's scientific rationale, research objectives and potential impact.

## Cardio‐metabolic resilience to obesity

According to the latest data from the Non‐Communicable Diseases Risk Factor Collaboration (2017), the number of adults worldwide with obesity increased from approximately 100 million to 671 million between 1975 and 2016, with an additional 1.3 billion in the overweight range. While this obesity epidemic is associated with a wide range of adverse mental and physical health conditions and societal costs (Withrow & Alter 2011; GBD 2015 Obesity Collaborators *et al*. 2017), individuals with obesity do not form a single homogenous group with the same health and disease risks. Indeed, it has been estimated that in 2015 nearly 40% of global deaths due to common non‐communicable diseases occurred in individuals who were not obese according to a body mass index (BMI) greater or equal to 30 kg/m^2^ (GBD 2015 Obesity Collaborators *et al*. 2017). This finding reflects the limitation of BMI in measuring adiposity and disease risk (Prentice & Jebb 2001; Flegal *et al*. 2009), but also illustrates that there is clearly substantial heterogeneity in cardio‐metabolic health among people with the same or at least similar BMI values.

Since the 1980s, the term metabolically healthy obesity (MHO) has been used to describe the sub‐group of individuals who are obese (normally according to BMI or waist circumference) but do not have any, or only minimal, cardio‐metabolic complications (typically including hypertension, dyslipidemia and hyperglycaemia). The idea that someone can be obese yet metabolically healthy has been highly controversial and widely debated (Piepoli *et al*. 2016; Stefan *et al*. 2018). A plethora of epidemiological studies have largely dispelled MHO as an unhelpful and misleading construct, namely because individuals with MHO (1) tend to develop cardio‐metabolic complications and transition to being unhealthy more frequently than their non‐obese counterparts (Bobbioni‐Harsch *et al*. 2012; Appleton *et al*. 2013; den Engelsen *et al*. 2013; Soriguer *et al*. 2013; Bell *et al*. 2015a,b; Hamer *et al*. 2015; Guo & Garvey 2016) and, therefore, (2) have greater incidence of various mortality and morbidity outcomes (*e.g*. coronary heart disease and type 2 diabetes) (Fan *et al*. 2013; Hinnouho *et al*. 2013, 2015; Aung *et al*. 2014; Gunter *et al*. 2015; Sung *et al*. 2015; Murphy *et al*. 2016; Caleyachetty *et al*. 2017; Lassale *et al*. 2018). As such, MHO is not a benign condition but rather an intermediate state before the development of cardio‐metabolic abnormalities. In many ways, it is also a man‐made artefact: categorising a population into sub‐groups by dichotomising measures, such as BMI and blood pressure, is nearly always going to identify a relatively small group who are obese (BMI ≥30 kg/m^2^) but do not have hypertension (blood pressure <140/90 mmHg), for example. However, this group with so‐called ‘healthy obesity’ is always going to have higher average blood pressure than the much larger group who are not obese (BMI <30 kg/m^2^) and do not have hypertension (blood pressure <140/90 mmHg). Such inherent baseline differences in risk factors between the healthy obese and healthy non‐obese groups are reported in the descriptive statistics table of most papers on this topic and are largely responsible for known differences in mortality risk between these two groups (Johnson *et al*. 2018b). Documented associations of MHO with disease or mortality risk might, therefore, be viewed as an artefact.

As surmised in a recent commentary (Johnson 2018), variation in cardio‐metabolic health and disease risk at any given BMI value (or even waist circumference *etc*.) is undeniable, but the concept of MHO is a crude way of capturing this heterogeneity. A lot of work over the decades has looked forward in time, finding and using somewhat obvious results (*i.e*. the most obese and unhealthy have the worst prognosis, the most normal weight and healthy have the best prognosis, and any group in between has intermediate prognosis) to argue that healthy obesity doesn't really exist. By contrast, very few studies have looked back in time and modelled the life course processes and exposures that explain why some people develop a disease or die while other people with the same BMI do not.

## Body size trajectories and life course exposures

The seminal publication of Abraham *et al*. (1971) was one of the first studies to investigate the consequences of early‐life characteristics for adulthood cardiovascular disease, followed closely, of course, by the pioneering work of Barker *et al*. (1989). By collecting long‐term follow‐up data on ~700 men who attended schools in Hagerstown, MD, US, between 1923 and 1928 and had weight recorded at the age of 9–13 years, Abraham *et al*. (1971) were able to show that rates of some cardiovascular diseases were highest among the group of individuals who were most overweight in adulthood but below average weight in childhood. This type of analysis, based on simple cross‐tabulations, also tells us about the childhood to adulthood trajectory that incurred the greatest disease risk: morbidity was greatest in overweight men who were below average weight children, so the greatest disease risk occurred in individuals who gained the most weight or had the steepest trajectory between childhood and follow‐up, 35–40 years later. These results essentially represent a statistical interaction, in which the well known positive association of adulthood obesity with cardiovascular disease risk is modified by childhood weight status. That is, the cardio‐metabolic complications of adulthood obesity were less pronounced for individuals who were also overweight in childhood, thereby inadvertently demonstrating how high childhood BMI might provide some degree of cardio‐metabolic protection against the adverse consequences of obesity in adulthood. Subsequent publications that have replicated this type of analysis have not typically discussed their findings within the context of healthy obesity (Juonala *et al*. 2011; Park *et al*. 2013; Bjerregaard *et al*. 2018), but from this literature it is clear that high childhood BMI is not a powerful disease risk factor in itself because the majority of morbidity and mortality occurs in adults who were normal weight children (Llewellyn *et al*. 2016), in agreement with the Abraham *et al*. (1971) findings.

Based on their work of the adiposity rebound, Rolland‐Cachera and Peneau (2013) have proposed that there are two main BMI trajectories that lead to adulthood obesity. The first is characterised by being big at all ages due to healthy levels of both fat and fat‐free mass, while the second is characterised by low or normal BMI in infancy, an early‐adiposity rebound, and subsequently an unhealthy level of fat accumulation. The first type of trajectory has been hypothesised to be associated with MHO in adulthood, while the second is clearly more deleterious and, as supporting evidence, is more characteristic of individuals born during (compared to before) the obesity epidemic era (Johnson *et al*. 2012a,b, 2013b, 2015). Few studies have, however, investigated how different BMI trajectories might lead to obesity but incur different health and disease risks. A recent paper using data from the 1966 *Northern Finland Birth Cohort* related BMI trajectories (summarised by timing and magnitude of infant BMI peak and adiposity rebound) to weight and health status at 31 years of age, but likely found spurious results because of the very low number of MHO individuals in their sample (9 men; 22 women) (Nedelec *et al*. 2018). Matta *et al*. (2016) have shown that being born with a birthweight appropriate‐for‐gestational age (AGA) confers a higher probability of adulthood MHO than being born small‐for‐gestational age (SGA), in line with the hypothesis of Rolland‐Cachera and Peneau (2013). Further, Johnson *et al*. (2017) have demonstrated that higher infant BMI might offer protection against the deleterious effects of adulthood obesity because of gains in fat‐free mass. This is supported by the study of Bouhours‐Nouet *et al*. (2008) that found higher infant weight gain to protect obese children (*n* = 117) from truncal adiposity and insulin resistance, which is intriguing given the evidence linking rapid infant weight gain to increased obesity risk (Druet *et al*. 2012). Beyond infancy, Salonen *et al*. (2009) found higher BMI at ages 2–11 years to protect obese adults (*n* = 499) from the metabolic syndrome, whereas Howe *et al*. (2014) found no robust evidence that rapid BMI gain between 7 and 13 years was protective for cardio‐metabolic health in obese Danish men (*n* = 284). The two studies that have investigated early‐onset obesity also reported equivocal results (Brochu *et al*. 2001; Howe *et al*. 2014). In summary, this literature base is small and inconclusive, with most studies having a limited sample size and focusing on just one body size trait. Perhaps the most reliable, consistent evidence is that being obese for a longer duration and to a greater intensity predicts being metabolically unhealthy (Mongraw‐Chaffin *et al*. 2016; Zamrazilova *et al*. 2016).

Body size trajectories are, of course, an obvious starting point for exploring the life course processes that might provide cardio‐metabolic resilience to obesity, and one study has augmented this work by looking at pubertal timing. In that paper, Bleil *et al*. (2012) observed that an earlier age at menarche was associated with increased odds of metabolically unhealthy obesity among 989 women aged 25–45 years, again tying in with the hypothesis of Rolland‐Cachera and Peneau (2013) because advanced pubertal development is related to an earlier adiposity rebound and childhood obesity (Ahmed *et al*. 2009; Johnson *et al*. 2013a; German *et al*. 2015). But, in order to intervene and prevent or delay the cardio‐metabolic complications of obesity, modifiable targets that are more tangible than BMI trajectory and pubertal timing are needed. The problem is that, while the cross‐sectional correlates of MHO are extensively documented, we know almost nothing about which, and how, life course exposures (operating during gestation, early life and beyond) explain heterogeneity in health and disease risk at any given BMI value (Phillips 2017; Robson *et al*. 2018). Such information would not only be useful in understanding which obese individuals have the lowest morbidity and mortality risks, but also in understanding which non‐obese individuals have the highest.

## Current and future research objectives

The aim of the UK Medical Research Council (MRC) New Investigator Research Grant (MR/P023347/1) ‘Body size trajectories and cardio‐metabolic resilience to obesity in three United Kingdom birth cohorts’ is to reveal the body size trajectories and pubertal development patterns that might attenuate the positive associations of adulthood obesity with cardio‐metabolic disease risk factors, thereby providing cardio‐metabolic resilience to obesity. This work will also quantify the trajectories and patterns that might result in some normal weight adults having poor health prospects. The focus will be on secondary data analysis in nationally representative UK birth cohort studies which have life course body size data and a biomedical sweep in adulthood, namely the MRC *National Survey of Health and Development* (1946 *NSHD*; Wadsworth *et al*. 2006), the *National Child Development Study* (1958 *NCDS*; Power & Elliott 2006) and the *British Birth Cohort* (1970 *BCS*; Elliott & Shepherd 2006). As part of the *Cohort and Longitudinal Studies Enhancement Resources* (*CLOSER*) initiative, the Principle Investigator (WJ) has already harmonised the body size data across these (and other UK) birth cohorts studies to investigate secular trends in obesity development (Johnson *et al*. 2015), socio‐economic inequalities in childhood and adulthood body size (Bann *et al*. 2017, 2018) and the contribution of infant weight gain to the adolescent obesity epidemic (Johnson *et al*. 2018a). This new grant is a continuation of this work, augmenting the harmonised body size data set with adulthood cardio‐metabolic measures.

Rather than having to define MHO, which is an inherently limited construct, the analysis plan focuses on developing models that treat continuous variables as continuous and estimate how the relationship of adulthood BMI with some risk factors (*e.g*. HbA1c) is modified by body size trajectories of pubertal development patterns. Using this type of interaction nods back to the Abraham *et al*. study (1971). Briefly, cardio‐metabolic risk factors will be considered separately and in composite outcome variables. Body size trajectories will be captured by (1) conditional change measures (that represent starting size and rate of change during different age periods), (2) traits from multilevel growth curve models (*e.g*. area under the BMI trajectory, length of time obese and age of obesity onset) and (3) latent trajectory classes from mixture models (Johnson 2015). Pubertal development patterns will be captured by individual as well as composite variables. Models will be built in each cohort study separately, and where appropriate meta‐analysis techniques will be used to pool estimates and investigate between‐study heterogeneity. Structural equation modelling will be used to determine the direct and indirect paths between body size trajectories, patterns of pubertal development and adulthood BMI and cardio‐metabolic risk. Further work utilising the sequential nature of the cohorts (*i.e*. 1946, 1958 and 1970) will quantify, and describe the contribution of between‐cohort differences in body size trajectories and pubertal development patterns to, any secular trend in cardio‐metabolic resilience to obesity.

The grant will also be used as a springboard to develop a much larger and comprehensive programme of research in this field. Work using the *English Longitudinal Study of Ageing* (Steptoe *et al*. 2013) to investigate how the MHO‐mortality association strengthens when the referent group comprises stable healthy non‐obese individuals identified from repeat biomedical assessments has already been published (Hamer *et al*. 2017), as has work using the *Whitehall II study* (Marmot *et al*.^1991^) to show how the MHO‐mortality association attenuates after accounting for inherent baseline differences in risk factors (Johnson *et al*. 2018b). Mortality data are also available in the *NSHD*,* NCDS* and *BCS* and, in collaboration with colleagues at University College London (UK), there are plans to link these data to the *CLOSER* harmonised body size data set for epidemiological investigation. The *NSHD* arguably has the richest life course and biomedical data, and a PhD student is working with WJ at Loughborough University to investigate how early‐life adversity and lifestyle behaviours might provide cardio‐metabolic resilience to obesity in this cohort. Other regional birth cohort studies in the UK have more frequent body size data in childhood (*e.g*. the *Avon Longitudinal Study of Parents and Children;* Boyd *et al*. 2013), providing the opportunity to model fine‐tuned trajectories that lead to adulthood obesity but have different cardio‐metabolic profiles. Inside and outside of the UK, there are also cohorts that have the necessary data to model which and how exposures explain heterogeneity in the consequences of other anthropometric indicators of disease risk (*e.g*. rapid infant weight gain). Finally, while this evolving research programme will naturally lend itself to the use of longitudinal birth cohort data, large‐scale survey data could be used to describe worldwide and temporal variation in BMI‐risk factor associations.

## Knowledge exchange and impact

Body size trajectories have played a central part in the evolution of life course epidemiology, and this grant is the next step in refining our understanding of their role in disease development. The results will constitute a significant advancement in our understanding of how, when and in whom some degree of cardio‐metabolic resilience to obesity develops. We envisage that the findings will inform on‐going research, including trials that aim to alter body size trajectories and stimulate new lines of investigation. This grant is also potentially relevant to a wide range of clinicians. Child growth assessment is a fundamental part of routine care in the UK and, by providing a better understanding of the role of growth and development in cardio‐metabolic disease aetiology, our findings have the potential to impact on this practice and the guidelines on what constitutes healthy growth and development. Obesity clinics and cardiologists alike may also benefit from an improved understanding of which patients are likely to suffer most from the cardio‐metabolic complications of obesity, based on their body size trajectory and other life course exposures. Findings could also potentially be used to augment recommendations to parents and their children on how to maximise long‐term cardio‐metabolic health. Of course, the end goal of this grant is to provide a stronger literature base to inform policy and practice, as a means to impact on public health and wellbeing. In particular, the results may be informative for characterising which individuals should be targeted for healthcare provision or therapeutic intervention and may be exploited for new evidence‐based interventions that improve disease processes.

To ensure maximum coverage and impact on the scientific and medical community, the results will be disseminated in publications and at international conferences. A collaborative group has been formed, and their steering and mentorship will naturally increase the impact of these outputs. Further, planned communication with organisations such as Public Health England will help ensure that the results are translational. A well‐thought‐out series of three workshops has been designed to provide training (*e.g*. to UK PhD students and postdocs on ‘cross cohort analysis of secular trends in longitudinal processes and relationships’) and increase dissemination and discussion of results. The grant is fully integrated into *CLOSER* and the institutions who house the studies, which will help (1) WJ strengthen his link with the UK birth cohorts, (2) facilitate communication with the wider academic and non‐academic community and (3) hopefully benefit the end‐user of birth cohort study data (*e.g*. by feeding back harmonised data sets and guides to key stakeholders). Finally, the grant incorporates numerous training and learning opportunities for the Principal Investigator and employed Research Associate and, as such, will have significant impacts on their career trajectories.

## Conclusions

Individuals who are obese have different cardio‐metabolic health and disease risks, as do individuals who are normal weight, but very little is known about the life course exposures and processes that explain this heterogeneity. Primarily using data from the UK birth cohort studies, this grant will provide novel evidence on the body size trajectories, patterns of pubertal development, and other factors (*e.g*. early‐life adversity) that lead to some people developing a cardio‐metabolic complication or disease or dying while other people with the same BMI do not.

## Conflict of interest

The author reports no conflict of interest.
